# Specific measures for data-intensive health research without consent: a systematic review of soft law instruments and academic literature

**DOI:** 10.1038/s41431-023-01471-0

**Published:** 2023-10-17

**Authors:** Julie-Anne R. Smit, Menno Mostert, Rieke van der Graaf, Diederick E. Grobbee, Johannes J. M. van Delden

**Affiliations:** Julius Center for Health Sciences and Primary Care, University Medical Center Utrecht, Utrecht, The Netherlands

**Keywords:** Medical research, Social sciences

## Abstract

It is a common misunderstanding of current European data protection law that when consent is not being used as lawful basis, the processing of personal data is prohibited. Article 9(2)(j) of the European General Data Protection Regulation (GDPR) permits Member States to establish a legal basis in national law that allows for the processing of personal data for scientific research purposes without consent. However, the European legislator has formulated this “research exemption” as an opening clause, rendering the GDPR not specific as to what measures exactly are required to comply with the research exemption. This may have significant implications for both the protection of personal data and the advancement of data-intensive health research. We performed a systematic review of relevant soft law instruments and academic literature to identify what measures are mentioned in those documents. Our analysis resulted in the identification of four overarching themes of suggested measures: organizational measures; technical measures; oversight and review mechanisms; and public engagement and participation. Some of the suggested measures do not substantially contribute to the clarification of the GDPR’s “suitable and specific measures” requirement because they remain vague or broad in nature and encompass all types of data processing. However, the themes oversight and review mechanisms and public engagement and participation provide valuable insights which can be put to practice. Nevertheless, further clarification of the measures and safeguards that should be installed when invoking the research exemption remains necessary.

## Introduction

Over the past decades, the importance of obtaining consent in medical research settings has been strongly emphasized [1]. However, in data-intensive health research it is often regarded impracticable or impossible to obtain (meaningful) consent [2–5]. A common misunderstanding of current European data protection law is that when consent is not being used as lawful basis, the processing of that persons data is prohibited [6]. While obtaining consent is a way to secure legitimate data processing, it is not the only way. Article 6 of the General Data Protection Regulation (GDPR) contains six legal bases for the processing of personal data, of which consent is one.

Processing personal data for health research purposes most likely involves “special categories” of personal data. The European legislator has labeled genetic data, biometric data and data concerning health—among others—as special categories of data [7], which merit a higher form of protection [7]. As a result, the processing of special categories of data must have a lawful basis as outlined in Article 6 of the GDPR, as well as fall under one of the ten exemptions listed in Article 9(2) GDPR.

The “research exemption” can be found under Article 9(2)(j) GDPR and allows for the processing of special categories of personal data if the processing is deemed necessary for scientific research purposes. In addition, it is required that the processing is in accordance with Article 89(1) GDPR and that it is based on Union or Member State law. Article 89(1) GDPR states that “*technical and organizational measures*” should be in place which “*may include pseudonymization*”. As such, Article 9(2)(j) GDPR contains an “opening clause”: Member States have been given the discretion to implement the research exemption into their national legislation. When they do so, it is required to provide for *“suitable and specific measures to safeguard the fundamental rights and interests of the data subject*” [7]. However, the GDPR does not provide much substance as to what constitutes suitable and specific measures.

Recent research has shown that the conditions in and the extent to which processing of health data for scientific research is allowed without consent differs between the Member States [8], and the many documents with the purpose of guiding policy in this area contain dissimilar terminology and concepts [9]. The fragmentation of data protection standards for scientific health research across the EU leaves researchers with a confusing legal landscape to maneuver [10, 11]. Additionally, concerns have been raised about the possible emergence of a disparity between legal requirements and ethical standards [12].

The lack of clarity regarding the measures that should be implemented when invoking the research exemption may harm the protection of personal data as well as hinder progress in data-intensive health research. To address this, we conducted a systematic review of soft law instruments and academic literature. Our goal was to identify the measures outlined in documents regarding the processing of personal data for health research purposes without consent. These documents contain valuable opinions and suggestions on how to ensure legally and ethically sound data processing when consent by the data subject is lacking. Moreover, the pace of publication of soft law instruments and academic papers is a lot higher than the trajectory of issuing official legal texts. Therefore, the measures and safeguards referred to in those documents provide us with a more up-to-date reflection of the current data-intensive scientific research climate. With this review we aim to contribute to substantiating the GDPR’s requirement of installing suitable and specific measures when invoking the research exemption in Article 9(2)(j) GDPR.

## Methods

To ensure complete and transparent reporting of the methods used, we based our review on the PRISMA-Ethics Reporting Guideline for Systematic Reviews on Ethics Literature [13]. Textual analysis and coding of the included soft law instruments and articles was achieved using NVivo 12 qualitative data analysis software. To conduct a thematic analysis, the authors retrieved quotes from all the included documents containing recommendations and/or opinions regarding measures that should be installed when health data are processed for scientific research purposes without consent. Each quote was assigned one or more codes, and an inductive approach was used to identify different overarching themes arising from the reviewed documents.

For the purpose of this systematic review, the term soft law is used to denote (international) declarations, guidelines, recommendations, frameworks and other documents that are not legally binding but that have an influence on the regulation of health research. Relevant soft law instruments were identified using the *International compilation of human research standards* (2020), a collection of laws, regulations and guidelines governing research from 133 countries and a number of international and regional organizations. We reviewed instruments that were included under *Guidelines* in the categories *International* and *Europe Regionwide*.

First, all instruments containing any guidance on processing personal data for scientific research were selected for review. This resulted in a list of 22 instruments. To ensure its comprehensiveness and to complement it if necessary, the list was reviewed by our academic and consortium partners with expertise in health law and research ethics. Ultimately, we included the instruments from this list that were: related to the GDPR’s territorial scope; specifically referring to the absence of consent and/or describing types of scientific research for which obtaining consent is impossible; mentioning measures that should be installed in such a situation. Exclusion criteria were: only describing legislations of non-EU countries; solely factually reflecting current legal policies without adding study results, views, opinions, reflections and/or suggestions for appropriate measures and safeguards; not written in English.

The academic literature was identified through a systematic search in PubMed and Embase. The queries were adjusted to the type of database (see Appendix 1 and 2). The initial search was performed on 02 Dec 2020 and produced 977 results in PubMed and 436 in Embase. After deduplication 1010 articles remained. Title/abstract screening left 250 articles remaining for full-text screening. An additional search with the same queries was performed on 24 Jan 2022. The additional search produced 148 results in PubMed and 97 in Embase. After deduplication 194 remained for title/abstract screening, of which 24 articles were included for full-text screening (see Table 1).

**Table 1:**
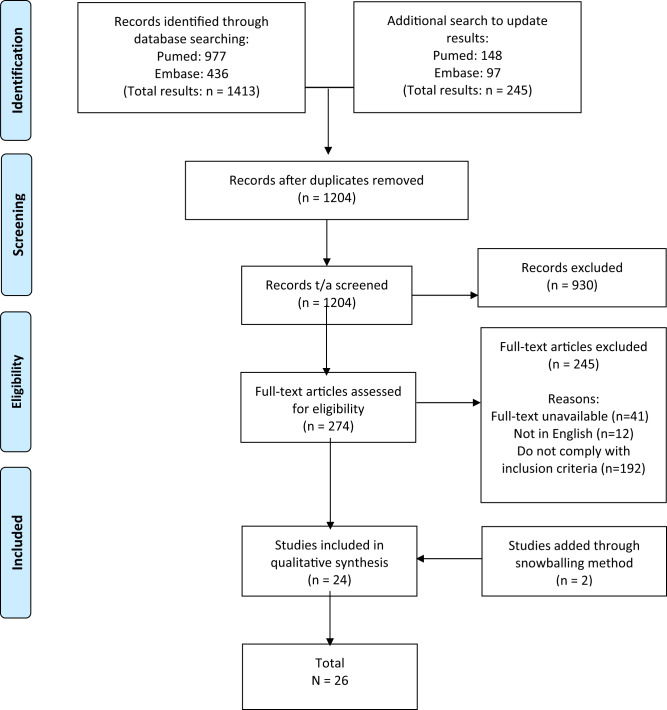
PRISMA flow chart.

Inclusion criteria were: academic publications related to the GDPR’s territorial scope; specifically referring to the absence of consent and/or describing types of scientific research for which obtaining consent is impossible; mentioning measures that should be installed in such a situation. Exclusion criteria were: only describing legislation of non-EU countries; solely factually reflecting current legal policies without adding study results, views, opinions, reflections and/or suggestions for appropriate measures and safeguards; not written in English. Publications were considered to be of sufficiently high quality if they were published in an international peer-reviewed journal. The screening of the articles was performed by two separate assessors (J.S. and M.M.). Disagreements regarding the eligibility of articles were resolved by close deliberation and consensus between the two separate assessors.

## Results

A total of 13 soft law instruments (see Table 2) and 26 scientific articles (see Table 3) were included, mentioning measures for processing health data for research purposes without consent. The thematic analysis of the quotes that were retrieved from the included soft law instruments and academic literature resulted in the identification of four overarching themes of suggested measures: *organizational measures, technical measures, oversight and review mechanisms*, and *engagement and participation*. Table 4 displays the literal wording of the retrieved quotes, along with their associated overarching themes.

**Table 2:** Included soft law instruments.

	Issuing authority	Document title	Year of issue
1.	Council for International Organizations of Medical Sciences (CIOMS)	International Ethical Guidelines for Health-related Research Involving Humans	2016
2.	European Data Protection Board (EDPB)	Guidelines 05/2020 on consent under Regulation 2016/679	2020
3.	European Data Protection Board (EDPB)	Guidelines on Transparency under Regulation 2016/679	2017
4.	European Data Protection Supervisor (EDPS)	Preliminary Opinion 8/2020 on the European Health Data Space	2020
5.	European Data Protection Supervisor (EDPS)	Preliminary Opinion on data protection and scientific research	2020
6.	Global Alliance for Genomics and Health (GA4GH)	Framework for Responsible Sharing of Genomic and Health-Related Data	2014
7.	World Medical Association (WMA)	Declaration of Helsinki—Ethical Principles for Medical Research Involving Human Subjects	2013
8.	World Medical Association (WMA)	Declaration of Taipei—Ethical Considerations Regarding Health Databases and Biobanks	2016
9.	International Society for Biological and Environmental Repositories (ISBER)	Best Practices: Recommendations for Repositories	2018
10.	Nuffield Council on Bioethics	The Collection, Linking and Use of Data in Biomedical Research and Health Care: Ethical Issues	2015
11.	Organisation for Economic Co-operation and Development (OECD)	Guidelines on Human Biobanks and Genetic Research Databases	2009
12.	Organisation for Economic Co-operation and Development (OECD)	Recommendation of the Council on Health Data Governance	2019
13.	United Nations Educational, Scientific and Cultural Organization (UNESCO)	International Declaration on Human Genetic Data	2003

**Table 3:** Included scientific articles.

	References	Paper type	Scope of paper	Aim of paper
1.	Bak et al. [16]	Review	Ethical aspects of data protection in Sudden Cardiac Arrest setting	To thematically analyze ethical aspects of non-interventional emergency medicine and critical care research.
2.	Becker et al. [47]	Viewpoint	Legal grounds and derogations in the GDPR for research during a pandemic	To help research institutions navigate European data protection law within the COVID-19 crisis.
3.	Boyd [50]	Commentary	Linking health data with census data on ethnicity	To reflect on the benefits and potential harms of linking health data with census data on ethnicity.
4.	Brown et al. [49]	Original article	Secondary use of health data without consent	To analyze the legality of the use of electronic patient records in the NHS for research without explicit patient consent under UK and EU law.
5.	Casteleyn et al. [51]	Research article	Ethics and data protection in environmental health studies using biomarkers	To summarize the main features of ethics and data protection in studies using biomarkers in the field of environmental health and to highlight current discussions on related questions and bottlenecks.
6.	Hansson [35]	Review	Ethical issues in biobank research	To review the literature regarding some major themes in the discussion about ethics and biobanks.
7.	Hill et al. [38]	Research article	Consent to secondary use of health data	To determine the range of public opinion about the use of existing medical data for research and to explore views about consent to a secondary review of medical records for research.
8.	Holm and Ploug [33]	Symposium article	Big Data and health research governance	To describe the current Danish system, to outline a likely development in the near future and to discuss whether the current Danish governance system for the secondary use of health data is still suitable.
9.	Larson et al. [22]	Original research—Special report	Clinical data for AI applications	To propose an ethical framework for using and sharing clinical data for the development of artificial intelligence applications.
10.	Laurie et al. [19]	Article	Governance of health research	To provide an overview of essential elements of good governance of data linkage for health-related research, to consider lessons learned so far and to examine key factors currently impeding the delivery of good governance in this area.
11.	Laurie and Sethi [34]	Article	Governance of health research	To assess and advocate a principles-based approach contrasting this with traditional rule-based approaches and to propose a model of principled proportionate governance.
12.	McGraw et al. [18]	Article	Patient privacy in pragmatic clinical trials	To explore both the ethical foundation and regulatory framework intended to protect privacy in pragmatic clinical trials and to review examples of novel approaches to respecting persons in research that may have the added benefit of honoring patient privacy considerations.
13.	Mostert et al. [3]	Policy	Big Data in medical research	To review how the dominant “consent or anonymize approach” is challenged in a data-intensive medical research context, and to discuss possible ways forwards within the EU legal framework on data protection.
14.	Parkin and Paul [24]	Research report	Secondary use of health data	To explore public views about the use of medical information for the post-marketing surveillance of medicine safety.
15.	Porsdam Mann et al. [23]	Discussion	Research ethics	To examine the ethical tensions that arise between the conflicting goals of advancing biomedical research and protecting patient privacy and to propose a risk-adapted framework for the facilitation of ethical uses of health data for the benefit of society.
16.	Price and Cohen [21]	Review article	Big Data	To outline the legal and ethical challenges big data brings to patient privacy.
17.	Richter et al. [46]	Article	Secondary use of clinical data	To examine whether abolishing consent for secondary data use would be acceptable to patients.
18.	Rumbold and Pierscionek [39]	Debate	GDPR	To examine and compare data protection laws in seven different jurisdictions governed by the GDPR.
19.	Schmit et al. [17]	Original paper	Patient communication	To improve communication with patients and transparency about how complex software, such as MiNDFIRL, is used to enhance privacy in secondary database studies to maintain the public’s trust in researchers.
20.	Shabani and Borry [36]	Review article	GDPR	To explore the major provisions of the GDPR with regard to processing genetic data, and to assess the influence of such provisions on reinforcing the legal safeguards when sharing genetic data for research purposes.
21.	Shabani et al. [37]	Review article	Genomic data sharing	To review oversight practices by Research Ethics and Data Access Committees and argue that they reveal a compelling need to clarify the scope of ethical considerations by oversight bodies and to delineate core elements such as “objectionable” data uses.
22.	Stjernschantz Forsberg et al. [48]	Analysis	Individual consent in biobank research	To argue that requiring informed consent for research on stored tissue samples and associated data not only defeats the interest of society but also runs counter to the interests of the individuals it purports to protect.
23.	Staunton et al. [4]	Policy	GDPR	To review soft legal tools, international treaties and other legal instruments that regulate the use of health research data.
24.	Thorogood and Zawati [15]	Symposium article	Genomic biobanking	To review international privacy norms governing human genomic biobanks and databases.
25.	Ulrich et al. [52]	Article	Patient privacy and clinical research	To discuss the ethical challenges of balancing patient privacy with advancing clinical research and ask what level of privacy and confidentiality can and should patients expect from their clinician providers, fellow research colleagues, and institutions.
26.	Williams and Pigeot [2]	Opinion paper	Ethical requirements for research	To critically discuss conventional approaches to research ethics that emphasize consent and data protection.

**Table 4:** Identified overarching themes.

	**Organizational measures**
Soft law	Transparency [44]
	To be transparent, fair and accountable [45]
	Limiting data access e.g., trough safe havens, TTP’s [14]
	Limiting data use e.g., trough formal agreements [14]
	Individuals should be provided with reasons for not honoring data processing objections or requests [20]
	Individuals should be able to express preferences regarding the processing of their personal health data [20]
	Informed consent [43]
	Accurate logging and auditing [25]
	A comprehensive security policy, organization and infrastructure, including both organizational and state-of-the- art technical security measures [25]
Literature	Transparency [15, 19, 39]
	Promote transparency and trust [17]
	Broader openness and accountability [2]
	Accountability [15]
	Extensive governance [15]
	Responsible data governance [16]
	Increased transparency regarding data protection and governance, as well as regarding research objectives [46]
	Clear and transparent policies on a multitude of issues [4]
	Clear and transparent governance procedures that oversee the use of data [4]
	Patients are made aware of how their data may be used [22]
	Individual notification [18]
	Allow individuals and the public to access clear information about the use of their data and their rights concerning this usage [3]
	Provide individuals with sufficient information and control over their data [3]
	Have patient representatives involved in crucial decisions about how their data will be used [21]
	Soliciting the attitudes of the involved parties regarding the associated risks [36]
	Specific targeted information provision [38]
	Training of personnel [47]
	Imposition of duties of confidentiality [47]
	Offering an opt-out mechanism [18]
	Introducing opt-out mechanisms before data collection [46]
	Adhere to relevant legal provisions [24]
	Inform health professionals about the outcomes of REC approved research [24]
	Researchers must ensure that their research proposals are trustworthy and reasonable [2]
	Take into account the pertinent individual or social concerns that may not be explicitly outlined in the legal provisions [36]

	**Technical measures**
Soft law	Data minimization, anonymization and data security [44]
	The use of privacy enhancing technologies [25]
	A comprehensive security policy, organization and infrastructure, including both organizational and state-of-the- art technical security measures [25]
Literature	Data security [15, 16]
	Individual privacy is carefully safeguarded [22]
	Data are aggregated when used for research and development [22]
	Proportionate technical measures [3]
	The use of IT and participant interfaces [3]
	The data should be key-coded [24]
	Encryption, pseudonymization, minimization of sensitive data processed [47]
	Using data that are de-identified to the fullest extent compatible with research aims [23]
	The use of safe houses, distributed databases and best practice in data management [23]
	Downstream control over access to data and samples [15]

	**Oversight and review mechanisms**
Soft law	Research ethics committee [27, 28]
	Independent ethics committee [29]
	Research ethics committees or comparable oversight mechanisms [30]
	Research ethics committee or an appropriate authority [30]
	An authorized entity such as a research ethics committee [30]
	Competent bodies or institutions [26]
	Oversight committees authorizing access to data [14]
	Research Ethics Committees (RECs) and Data Access Committees [14]
	Independent, multidisciplinary and pluralist ethics committees [31]
	An authorized human subject/ethics committee [32]
	Ethics committees [25]
Literature	Oversight by the Research Ethics Committee or Data Protection Officer [16]
	A research ethical assessment of projects [33]
	Institutional oversight mechanisms [22]
	Authorization by research ethics committees [34]
	Authorization body [19]
	An independent necessity and proportionality test, for instance by an (data access) ethics committee [3]
	Research ethics committees [23]
	Ethical review boards [35]
	Competent oversight bodies such as ethics committees and data access committees [36]
	Coordinated and well-functioning oversight bodies [37]
	Both REC’s and DAC’s [37]
	Independent and interdisciplinary review and oversight [4]
	Institutional oversight that may include approval by an ethics committee or some other body [4]
	Approval by an ethics review board [48]

	**Public engagement and participation**
Soft law	Making public the results of such assessments [DPIA’s ed.] [25]
Literature	Public engagement [16, 19]
	Genuine engagement with stakeholders and public groups [19]
	Stimulate participation by relevant stakeholders [3]
	Continuing public engagement [39]
	Public education about research [38]
	Broad notification [18]
	Community consultation [18]
	Greater input into research and research policies [18]
	Public awareness about research approved by ethics committees [24]
	Inform the public about the outcomes of REC approved research [24]
	The public needs to be made aware of medical research without consent [49]
	The circumstances for medical research without consent need to be discussed and consensus formed as to when that should be permitted [49]
	Public outreach and education explaining the benefits of well-designed EHR-based research performed under stringent privacy protection [23]
	Provide evidence that the public in general and ethnic minority populations in particular not only have participated in fully informed discussion of the issues, but also that these discussions have led to positive approval of what is proposed [50]

### Organizational measures

The first overarching theme regards organizational measures. According to the Nuffield Council on Bioethics, when performing data-intensive health research without consent, “*additional* governance arrangements are usually required.” This could include limiting the use of data through formal agreements such as Data Sharing Agreements, Data Re-use Agreements and Material Transfer Agreements [14]. The term governance is referred to in multiple other documents as well: for instance, the requirement of *extensive* governance to ensure that secondary uses are legitimate (i.a.), for which the principles of transparency and accountability are vital [15]. Another example is the call for *responsible* data governance, in which the authors feel that data governance policies should not only aim to protect privacy but that they should also address broader societal issues such as fairness [16].

Of all different organizational measures that were mentioned, transparency was repeated most and emerged from the reviewed soft law documents as well as the scientific literature. The importance of clear and transparent policies regarding topics such as “data transfers, feedback of findings, storage of data, (..), re-contact of data subjects, access requests from third parties, access requests of data subjects, governance, and (where applicable) intellectual property and commercial use” was emphasized [4]. Furthermore, it was stated that by “adopting patient-friendly public disclosures relating to privacy safeguards and risks”, “describing how technology is used to safeguard participant data” and by providing “a privacy statement that increases database research transparency and discusses the software used to enhance privacy” trust and transparency will most likely be promoted [17].

It was argued that a form of respecting patients’ interests is through informing and notifying them [18], and that “for nonconsensual research to be defensible, broader openness and accountability must play an even greater role [2].” It was suggested that effectuating transparency can largely be achieved through publication on websites and social media [19]. Individual notification as well as broad notification through posters, emails, brochures, social media, or web portals were also proposed [18].

In addition, several documents emphasize that patients and/or individuals should be able to exert control over ‘their’ data, that they should be able to express their preferences regarding the processing [20] and that they should be involved in crucial decisions about how their data will be used [21].

### Technical measures

The second theme concerns technical measures that can be implemented for the protection of personal data and the rights of the data subject. Data security is regarded not just as an important safeguard against unauthorized access to data, but also against loss, destruction, and modification [15]. In multiple of the included scientific articles technical measures are mentioned in congruence with, or as a part of, a governance structure. For instance, some regard “security and oversight” as one of the main components of data governance [16]. In addition, others state that “proportionate technical and governance measures should be incorporated in the design of data-intensive medical research projects and infrastructures [3]”.

Examples of suggested technical measures are aggregating data [22], de-identifying data [23] and key-coding data [24]. In the preliminary opinion on the European Health Data Space (EHDS) by the European Data Protection Supervisor (EDPS)_it is stated that the use of effective encryption should be a baseline requirement for the incorporation of state-of-the-art technical security measures. Furthermore, this document provides in-depth guidance on what should be understood by the term privacy enhancing technologies. For instance, the opinion refers to technologies “enabling to perform operations on encrypted data without having access to the data in clear or performing calculations on distributed data without having access to all data sources or enabling reliable statistical calculations on data where noise has been injected [25]”.

### Oversight and review mechanisms

Thirdly, many of the reviewed documents state that there should be some form of oversight and/or review when performing data-intensive health research without consent by the data subject. Table 4 shows that different mechanisms are deemed suitable for the task of performing oversight and/or review. Some documents state that oversight or review should be performed by “competent bodies or institutions [26]” or an “authorization body [19]”. (Research) Ethics Committees (RECs) were most often suggested  [3, 4, 14, 16, 23, 27–37]. Moreover, several documents mention Data Access Committees (DACs) as the appropriate body for oversight or review [3, 14, 36, 37]. It was asserted that “RECs and DACs have a critical role to play in protecting the rights and interests of data donors and promoting the social value and public good of genomic data sharing [37]”.

Various characteristics were attributed to the designated mechanism for oversight or review such as “independent, multidisciplinary and pluralist [31]” or “coordinated and well-functioning [37]”. Furthermore, the importance of ensuring that oversight bodies have “adequate expertise” was stressed, meaning that they should possess sufficient knowledge about the processing of (genomic) data and the associated risks [36]. Some of the documents mention specific tasks and goals for the oversight or review mechanisms i.e., to “waive informed consent [28, 30, 32]”, “make an assessment of research proposals [36]”, “ensure that clinical data are used appropriately and only for purposes that will be beneficial to future patients [22]”, to perform “an independent necessity and proportionality test [3]” or to “address the requirements of adopting organizational measures and safeguards when processing personal data [..] [37].”

Multiple documents elaborate on the conditions under which the processing of personal data without consent should be permitted by the oversight or review body. Our analysis revealed that the conditions under which the consent requirement can be surpassed, vary significantly across different documents and/or authors. Often, the acceptability of surpassing the consent requirement is contextual and depends on the circumstances of a specific case. For instance, the World Medical Association’s (WMA) Declaration of Helsinki takes into account “exceptional situations where consent would be impossible or impracticable to obtain [27].” Alternatively, the Organization for Economic Co-operation and Development’s (OECD) Guidelines on Human Biobanks and Genetic Research Databases state that “in some jurisdictions, consent may be waived when it cannot be obtained, the risk to the participant is deemed minimal, and the rights and welfare of the participant are not adversely affected. In such cases, the informed consent may be waived by an authorized entity such as a research ethics committee in accordance with the applicable law and ethical principles pertaining to the protection of human subjects and will vary from jurisdiction to jurisdiction [30]”. Furthermore, the 2016 International Ethical Guidelines for Health-related Research Involving Humans of the Council for International Organizations of Medical Sciences (CIOMS) state that research ethics committees may approve a “waiver of informed consent to research if the research would not be feasible or practicable to carry out without the waiver, the research has important social value, and the research poses no more than minimal risks to participants [28].”

### Public engagement and participation

The final overarching theme concerns the engagement of the public and the participation of relevant stakeholders in the research process. The reviewed documents prominently show the importance of engaging the public and not just the data subject. Many included documents emphasize the importance of public engagement, community consultation and/or stakeholder participation. It has been stated that “increasing public education about research and specific targeted information provision could promote trust in research processes and safeguards, which in turn could increase the acceptability of research without specific consent [38].”

Many of the reviewed documents suggest that simply providing information about how the data is handled and its intended purposes is inadequate. It was argued that researchers and research institutions should strive for “genuine engagement with stakeholders and public groups” which could include “the possibility of influencing matters, including the direction of research where appropriate [19].” Reciprocity seems to become more important and therefore, continuing public engagement should be upheld “to ensure that the requirements for social license are fulfilled and the research community continues to deserve the trust of society [39].” One of the reviewed documents indicated that the involvement of stakeholders could complement the REC review and assist in legitimizing data research [16].

## Discussion

This systematic review of relevant soft law instruments and academic literature resulted in the identification of four overarching themes of measures for performing data-intensive health research without consent. The aim of this review was to contribute to substantiating to the GDPR’s requirement of installing *suitable and specific measures* when invoking the research exemption in Article 9(2)(j) GDPR.

One of the distinctive findings is that many of the reviewed documents recommend subjecting data-intensive health research without consent to review by a REC, DAC or a comparable review mechanism. In most European jurisdictions, obtaining research ethics approval for the (secondary) use of health data for research purposes is currently not a legal requirement [11]. Our research implies that in Member States where approval from an oversight or review mechanism is currently not legally required, proportionate review could be made part of the governance structure of health data research initiatives.

In its opinion on the proposed EHDS, which aims to not only to improve access to and quality of healthcare but also to support scientific research, the EDPS emphasizes the importance of ethical data use. The opinion highlights the value of ethics committees and advises that they are taken into account in forthcoming legislation [25]. The benefits of implementing oversight bodies in genetic research specifically are emphasized by the EDPS: “Genetic research in particular has implications not only for the subject of the DNA tests but others in his or her family or with shared characteristics in this and future generations. Independent ethical committees could support the understanding of which activities qualify as genuine research and define the ethical standards referred to in the GDPR [40].”

It appears that the European legislator has already incorporated the EDPS’ views in the design of the Data Governance Act (DGA), which will be applicable from September 2023, and is intended to regulate the re-use of data collected in public institutions. The DGA introduces the concept of data altruism, which is the voluntary disclosure of data by individuals or companies for the common good, including scientific research purposes. The European legislator asserts that for the concept of data altruism to succeed, safeguards such as oversight by ethics councils or boards will ensure that the data controller complies with high standards of scientific ethics [41].

Moreover, another role for oversight and review bodies could be to assist in the clarification of the role of consent in data-intensive scientific research. It seems that confusion has risen about the role of consent, because the term “consent” is being used in various regulatory areas without necessarily fulfilling the same purpose [42]. For instance, consent can be used as a legal basis for personal data processing, but it can also serve as an ethical standard and/or safeguard, providing individuals with more choice and control [6, 43]. These different forms and purposes of consent can also be found in the documents that were included for this review. For instance, the Declaration of Helsinki and the CIOMS guidelines (i.a.) contain ethical norms. When reference is made to consent in those documents, they refer to a different consent from the consent that is included in Articles 6 and 9 GDPR. According to the European Data Protection Board (EDPB) these different functions can and should be distinguished [7]. The EDPS is of the opinion that “viewing them as a single and indivisible requirement would be simplistic and misleading” [43]. Deliberations between the research community and data protection experts will be necessary to shape the notion of consent in the future of scientific research. Review and oversight bodies should be included in these deliberations.

Another notable result of our review is the identification of the theme *public engagement and participation*, which reveals emphasis on the importance of engaging the broader public in scientific research endeavors. Although the GDPR primarily focuses on the protection of the rights of the person whose data is being processed, most of the reviewed soft law instruments and, more prominently, the academic literature indicate that this is not sufficient. The majority of the included literature advocates informing the public, rather than solely informing the individual about (i.a.) data-intensive health research that is being performed without consent, the review processes by ethical oversight bodies, and the outcomes thereof. Furthermore, the reviewed literature seems to underline the importance of not just informing, but also actively involving and engaging the public, and thereby enabling them to genuinely participate in scientific research processes.

At the same time, some of the suggested measures identified in the reviewed soft law instruments and academic literature did not sufficiently clarify the GDPR’s requirement of installing *suitable and specific measures* when invoking the research exemption in Article 9(2)(j) GDPR. Many of the suggested measures included in the themes *technical measures* and *organizational measures* such as transparency, accountability, data-minimization and pseudonymization are a mere repetition of legal principles or standards deriving from the GDPR and are in in fact applicable to all types of data processing, including situations where consent has been obtained [7].

In addition, many of the reviewed documents recommend a certain measure, such as “transparency” or “data security”, without any further specification or clarification of what those terms constitute or what should be done to promote them. As such, it is unclear whether these documents and authors use the terms “transparency” and “data security” to refer to the same meaning of those terms as the GDPR does. Moreover, the implementation of measures should be proportionate to, for instance, the risks or the sensitivity of the data. However, in the reviewed documents little attention is paid to the proportionality of the suggested measures.

The lack of specification of a large part of the identified measures impedes the substantiating of the *suitable and specific measures* requirement when invoking the research exemption. Moreover, it complicates determining whether there indeed is a disparity between ethical and legal requirements. The EDPS has suggested that in the context of the EHDS a gap analysis might be required. This gap analysis will reveal whether there is a need to integrate with other regulatory safeguards provided by, for instance, ethical guidelines [25]. A similar gap analysis in the context of the GDPR could be of value.

This study has potential limitations. The results could be influenced by the exclusion of documents that were not available full text (see Table 1). Furthermore, it is possible that the search strategy used on the soft law instruments has resulted in the failure to identify all relevant documents. Moreover, by only including documents written in English with global and/or European relevance we might have missed valuable suggestions for specific measures included in, for example, national guidance documents. Future research endeavors could be aimed at exploring measures which are included in documents drafted for specific jurisdictions.

## Conclusion

This review has provided us with some valuable insights on how to substantiate the GDPR’s requirement of installing *suitable and specific measures* in accordance with Article 9(2)(j) GDPR. The results suggest that this could be done, for instance, by making review by a REC or DAC part of the governance structure of health data research initiatives. It is also proposed to inform and engage not only the data subjects, but also different stakeholders and the public regarding the use of health data for research purposes.

This research does not provide sufficient basis to conclude whether it is also desirable to translate the suggestions we have found into legal obligations. This review can provide inspiration, but the results will still need to be reflected on. The mere fact that something is mentioned in soft law instruments or in the academic literature does not necessarily mean it should be turned into law. It would have to be evaluated, for instance, whether the suggested measures can withstand a subsidiarity and proportionality test. Therefore, we strongly encourage the European legislator, the Member States and the EDPB and/or other international ethical/legal guidance committees to further clarify the *suitable and specific measures* requirement and issue more in-depth guidance on this subject.

### Supplementary information

Appendices pdf format

## Data Availability

All data generated or analyzed during this study are included in this published article and can be accessed through the search strings included in Appendix 1.
