# Supplementary material for: Specific measures for data-intensive health research without consent: a systematic review of soft law instruments and academic literature
Source: Eur J Hum Genet. 2023 Oct 17;32(1):21–30. doi: 10.1038/s41431-023-01471-0 (PMC10772063; doi:10.1038/s41431-023-01471-0)
Supplement: Supplementary file 1 — Appendices pdf format [file 41431_2023_1471_MOESM1_ESM.pdf]

Appendix 1. Search queries

Database	Query	Results (02/12/20)	(24/01/22)
PubMed	(data sharing[Title/Abstract] OR "Information Dissemination"[Mesh] OR data link*[Title/Abstract] OR data processing[Title/Abstract] OR secondary us*[Title/Abstract] OR health data reus*[Title/Abstract] OR data reus*[Title/Abstract] OR big data[Title/Abstract] OR "Big Data"[Mesh] OR electronic health record*[Title/Abstract] OR "Electronic Health Records"[Mesh] OR retrospective research[Title/Abstract] OR scientific research[Title/Abstract] OR health research[Title/Abstract] OR health data research[Title/Abstract]) AND (consent[Title/Abstract] OR informed consent[Title/Abstract] OR "Informed Consent"[Mesh] OR consent waiver[Title/Abstract] OR waiver of informed consent[Title/Abstract] OR consent exemption[Title/Abstract] OR research exemption[Title/Abstract]) AND (data protection[Title/Abstract] OR data security[Title/Abstract] OR "Computer Security"[Mesh] OR privacy[Title/Abstract] OR "Privacy"[Mesh] OR GDPR[Title/Abstract] OR general data protection regulation[Title/Abstract] OR measure*[Title/Abstract] OR safeguard*[Title/Abstract] OR governance[Title/Abstract])	977	148
Embase	('data sharing':ti,ab,kw OR 'data sharing'/exp OR 'data link*':ti,ab,kw OR 'data processing':ti,ab,kw OR 'secondary us*':ti,ab,kw OR 'health data reus*':ti,ab,kw OR 'data reus*':ti,ab,kw OR 'big data':ti,ab,kw OR 'big data'/exp OR 'electronic health record*':ti,ab,kw OR 'electronic health record'/exp OR 'retrospective research':ti,ab,kw OR 'scientific research':ti,ab,kw OR 'health research':ti,ab,kw OR 'health data research':ti,ab,kw) AND (consent:ti,ab,kw OR 'informed consent':ti,ab,kw OR 'informed consent'/exp OR 'consent waiver':ti,ab,kw OR 'waiver of informed consent':ti,ab,kw OR 'consent exemption':ti,ab,kw OR 'research exemption':ti,ab,kw) AND ('data protection':ti,ab,kw OR 'data security':ti,ab,kw OR 'computer security'/exp OR privacy:ti,ab,kw OR 'privacy'/exp OR gdpr:ti,ab,kw OR 'general data protection regulation':ti,ab,kw OR measure*:ti,ab,kw OR safeguard*:ti,ab,kw OR governance:ti,ab,kw)) AND [embase]/lim AND ('article'/it OR 'article in press'/it OR 'review'/it)	436	97

Appendix 2. Breakdown of search terms

**Data sharing**	**AND**	**Consent**	**AND**	**Data protection**
Data linking		Informed Consent		Data security
Data processing		Consent waiver		Privacy
Secondary use		Waiver of informed consent		GDPR
Health data reuse		Consent exemption		General data protection regulation
Data reuse		Research exemption		Measures
Big data				Safeguards
Electronic health records				Governance
Retrospective research				
Scientific research				
Health research				
Health data research				
